# Analysis of the Mediating Effect of Vitamin D via the TGF‐β1/Treg Pathway in the Pathogenesis of Childhood Primary Immune Thrombocytopenia

**DOI:** 10.1155/jimr/4001873

**Published:** 2026-03-26

**Authors:** Peiling Li, Mengru Chen, Zhihang Wang, Rui Fan, Jia Guo, Bao Liu, Zhiyin Wang, Yanyan Ma, Dongju Zhao, Shujun Li

**Affiliations:** ^1^ Department of Pediatrics, The First Affiliated Hospital of Xinxiang Medical University, Weihui, Henan, China, xxmu.edu.cn; ^2^ Department of Laboratory Medicine, The First Affiliated Hospital of Henan Medical University, Weihui, Henan, China

**Keywords:** immune thrombocytopenia, regulatory T cells, transforming growth factor-beta 1, vitamin D

## Abstract

**Objective:**

To investigate the role of vitamin D (VitD), transforming growth factor‐β1 (TGF‐β1), and regulatory T cells (Treg) in the pathogenesis of primary immune thrombocytopenia (ITP) in children.

**Methods:**

From February 2023 to September 2024, 51 children with ITP and 44 healthy children from the First Affiliated Hospital of Xinxiang Medical College were enrolled. The serum levels of VitD and TGF‐β1 and the percentage of Treg cells in peripheral blood were measured.

**Results:**

There was no significant difference in age and sex between the two groups (*p*  > 0.05). ITP group VitD, TGF‐β1, and the level of Treg cells were significantly lower than those of the control group (*p*  < 0.05). In the ITP group, VitD and TGF‐β1 (*r* = 0.421), Treg cells (*r* = 0.516), TGF‐β1 and Treg cells (*r* = 0.563), and platelet count (*r* = 0.399, 0.305, 0.361, respectively, *p*  < 0.05). The median model analysis showed that VitD had a significant negative overall effect on ITP risk (regression coefficient = −0.014, *p* = 0.004), but its direct effect was no longer significant after the introduction of TGF‐β1 and Treg, suggesting a complete mediation effect, where the path of VitD affecting ITP via TGF‐β1 is significant (effect value = −0.015, *p*  < 0.001), but the mediated pathway involving Treg was not statistically significant.

**Conclusion:**

There is dysregulation of VitD, TGF‐β1, and Treg cells in newly diagnosed children with ITP. TGF‐β1 may be a key mediator of the regulation of ITP by VitD, suggesting the potential value of TGF‐ β1 as an intervening target.

## 1. Introduction

Primary immune thrombocytopenia (ITP) in children is an immune‐mediated thrombocytopenic disease with an incidence of about 5–10 per 100,000 population, with an increasing trend with age [1]. The main clinical features are platelet count below 100 × 10^9^/L and a significant increase in bleeding tendency [2]. Although ~80% of children can achieve spontaneous remission within 1 year [3], some cases have progressed to chronic or refractory ITP, which seriously affects the patient’s quality of life and is even life‐threatening [4]. Currently, ITP’s pathogenesis has not been fully clarified, and the complex interaction between humoral immunity and cellular immunity jointly participates in the occurrence and development of diseases, and the specific molecular mechanism remains to be further studied [5].

The active metabolite 1,25‐(OH)_2_D_3_ of vitamin D (VitD) is involved in the regulation of various immune processes by binding to the VitD receptor (VDR) in the immune cell nucleus, including inhibition of Th1/Th17 cell‐mediated inflammatory response, reduction of production of autoantibodies by B cells, and promotion of regulatory T cell (Treg) proliferation and immunosuppressive function [6]. VitD deficiency has been shown to be closely associated with various autoimmune diseases [7], but its mechanism of action in idiopathic thrombocytopenic purpura is unclear. Treg cells are a key immunomodulatory subpopulation for the maintenance of immune tolerance, and their reduced number or dysfunction is considered an important immunological basis for the development of ITP [8, 9]. Transforming growth factor β1 (TGF‐β1) is a key cytokine to induce Treg cell differentiation. It promotes the differentiation of naive T cells to the Treg phenotype by upregulating Foxp3 expression, which is crucial for maintaining immune homeostasis [10, 11]. The study found that the level of TGF‐β1 and the proportion of Treg cells in children with ITP were significantly lower than those in healthy children, suggesting that the low level of TGF‐β1 may be an important cause of Treg dysfunction [12]. Therefore, VitD can promote the differentiation and functional recovery of Treg cells by regulating the expression of TGF‐β1. VitD is expected to be an important pathway for regulating the immune imbalance of ITP, and provides a theoretical basis for further revealing the pathogenesis of ITP and developing precise immune intervention strategies.

In this study, the differences of serum VitD level, Treg cell ratio, and TGF‐β1 concentration between children with ITP and healthy controls were analyzed retrospectively, and the relationship between them and platelet count was further explored. Based on the bootstrap mediation model, the potential mediating role of TGF‐β1/Treg regulatory network in the immune imbalance of VitD‐regulated ITP was further validated. The purpose of this study was to elucidate the key role of VitD in the pathogenesis of ITP and to provide theoretical support for mechanism exploration and precise immune intervention.

## 2. Materials and Methods

### 2.1. Study Participants

A retrospective analysis was conducted to screen children with thrombocytopenia (*n* = 68) admitted to the Department of Pediatric Internal Medicine of the First Affiliated Hospital of Xinxiang Medical College from February 2023 to September 2024. Strict inclusion and exclusion criteria were formulated according to the latest diagnostic criteria [13]. Inclusion criteria included the following: (1) age from 1 month to 16 years; (2) initial diagnosis and complete clinical data; and (3) no immunomodulatory therapy (e.g., IVIG, glucocorticoids, TPO receptor agonists, etc.). Exclusion criteria were (1) persistent (duration 3–12 months) or chronic ITP (duration >12 months); (2) secondary thrombocytopenia (including drug‐induced, autoimmune disorders, viral infections, hematopoietic neoplasms, and hereditary thrombocytopenia); (3) severe hepatic and renal insufficiency; (4) recent (within 3 months) immunosuppressive therapy; and (5) incomplete clinical data. After strict screening, 51 newly diagnosed children with ITP were included. The diagnosis of all cases was independently confirmed by two attending physicians and blood specialists with professional titles or above.

The control group included 44 healthy children who had physical examinations in our hospital at the same time. The criteria for inclusion were no recent history of infection, no use of glucocorticoids, and no treatment with blood products such as blood transfusion or gamma globulin. Age and gender matching ensured that the control group was well comparable to the case group in baseline characteristics.

This study was approved by the Medical Ethics Committee of the First Affiliated Hospital of Xinxiang Medical College (Approval Number EC‐023‐222).

### 2.2. Experimental Methods

Collect 4 mL of peripheral blood from all subjects into the coagulation‐promoting tube, centrifuge at the speed of 3000 r/min for 10 min, separate the serum, take 1 mL of serum into the cryo tube, and place it in −80°C refrigerator for freezing. It was used to detect the concentration of TGF‐β1, and the remaining serum was used to detect the level of VitD. An additional 2 mL of peripheral blood was collected into anticoagulant tubes for the determination of Treg cell percentage. The VitD level was detected by magnetic particle chemiluminescence with Autolumo A6000 (Zhengzhou Antu Bioengineering Co., Ltd.) (Kit Number 20252400176, Zhengzhou Antu Bioengineering Co., Ltd.); the concentration of TGF‐β1 was detected by enzyme‐linked immunosorbent assay (ELISA) using an EPOCH2 microplate reader (Burton Instruments Co., Ltd.) (Kit Number EH0287, Wuhan Fien Biotechnology Co., Ltd.); and the proportion of Treg cells was determined by flow cytometry using a DxFLEX flow cytometer (Beckman Coulter, Inc., USA). All experimental operations were performed in strict accordance with the kit instructions.

### 2.3. Qualification and Quality Control Statement

The assays in this study were performed by lead technicians from our institution, with over 15 years of experience. All researchers involved in sample processing and data analysis underwent standardized training and evaluation before the project. Rigorous quality control protocols were followed, including calibration of critical instruments, double‐checking key steps, and independent data entry and cross‐verification by two researchers to minimize errors and ensure reproducibility.

### 2.4. Statistical Processing

Statistical data analysis was performed using SPSS 26.0. Normal distribution measurement data adopt mean ± standard deviation (*x ± s*), indicating that the independent sample *t*‐test is used for the comparison between the two groups; nonnormal distribution data were expressed by median (interquartile range) [M (P25, P75)], and the Mann–Whitney *U* test was used for comparison between two groups. The count data were expressed by cases (*n* [%]), and the *χ* ^2^ test was used for comparison between the two groups; Pearson’s and Spearman’s correlation analyses were used. The total effect of VitD on ITP, the direct effect, and the indirect effect via the TGF‐β1/Treg pathway were analyzed using the bootstrap sampling method in R4.2.3, and each effect value was reported with its 95% confidence interval. Plot using Graphpad Prism 8.0 software. The difference was statistically significant (*p*  < 0.05).

## 3. Results

### 3.1. Comparison of Clinical and Laboratory Characteristics Between the ITP and Control Groups

**Figure 1:**
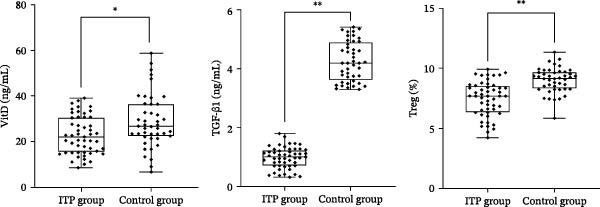
Comparison of VitD, TGF‐β1 levels, and Treg cells between the ITP group and the control group.  ^∗∗^ *p*  < 0.001;  ^∗^ *p*  < 0.05. Sample sizes: ITP group *n* = 51, control group *n* = 44.

In this study, there were 51 patients in the ITP group, 32 males and 19 females. Age: 1 month–180 month. There were 44 cases in the control group, 27 males and 17 females. The age ranged from 3 to 168 months. There was no significant difference in age (*z* = −0.231, *p* = 0.817) and sex (*χ* ^2^ = 0.019, *p* = 0.890) between the ITP group and the control group. There was no significant difference between the ITP group and the control group (*p*  > 0.05). Compared with the control group, the platelet count in the ITP group was significantly decreased, and the median platelet count in the ITP group and control group was 13.00 (4.00, 45.00) × 10^9^/L and 356.50 (285.75, 402.00) × 10^9^/L, respectively. The difference was statistically significant (*p*  < 0.001). The level of VitD was (22.77 ± 8.30) ng/mL in the ITP group and (28.87 ± 11.67) ng/mL in the control group (*p*  < 0.05). The levels of Treg cells and TGF‐β1 in the ITP group were significantly lower than those in the control group (*p*  < 0.001) (Table 1 and Figure 1).

**Table 1:** Comparison of clinical and laboratory characteristics between the ITP group and the control group.

Item	ITP group (*n* = 51)	Control (*n* = 44)	*χ^2^/z*	*p*
Age (months)	49.00 (23.00, 84.00)	45.50 (23.50, 87.00)	−0.231	0.817
Sex	—	—	0.019	0.890
Male	32 (62.7%)	27 (61.4%)	—	—
Female	19 (37.3%)	17 (38.6%)	—	—
WBC (×10^9^/L)	8.01 (5.83, 9.86)	7.63 (6.13, 9.27)	−0.866	0.387
RBC (×10^12^/L)	4.32 ± 0.49	4.36 ± 0.38	−0.384	0.702
Hb (g/L)	119.00 (111.00, 129.00)	120.50 (114.00, 125.75)	−0.224	0.823
PLT (×10^9^/L)	13.00 (4.00, 45.00)	356.50 (285.75, 402.00)	−8.376	<0.001
VitD (ng/mL)	22.77 ± 8.30	28.87 ± 11.67	−2.963	0.004
TGF‐β1 (ng/mL)	1.00 (0.71, 1.24)	4.19 (3.61, 4.90)	−8.375	<0.001
Treg (%)	7.47 ± 1.45	9.02 ± 1.04	−6.036	<0.001

*Note:* ITP, primary immune thrombocytopenia.

Abbreviations: PLT, platelet; RBC, red blood cell; TGF, transforming growth factor; WBC, white blood cell.

### 3.2. Correlation Between VitD, TGF‐β1, and Treg Cells in ITP and Control Groups

In the ITP group, serum VitD levels were positively correlated with TGF‐β1 concentrations (*r* = 0.421, *p* = 0.002) and with the proportion of Treg cells (*r* = 0.516, *p*  < 0.001), while Treg cell levels were also positively associated with TGF‐β1 levels (*r* = 0.563, *p*  < 0.001), as shown in Figure 2. Notably, Treg cells were significantly lower in children with ITP compared with healthy controls. In contrast, no significant correlations were observed in the control group between VitD and TGF‐β1 (*r* = 0.103, *p* = 0.504), VitD and Treg cells (*r* = 0.005, *p* = 0.972), or between Treg cells and TGF‐β1 (*r* = 0.009, *p* = 0.955).

Figure 2Correlation analysis of VitD, TGF‐β1, and Treg cells in the ITP group. (A), (B), and (C) represent the correlation between VitD and TGF‐β1, VitD and Treg cells, and Treg cells and TGF‐β1, respectively. Sample size: *n* = 51.

**Figure:**
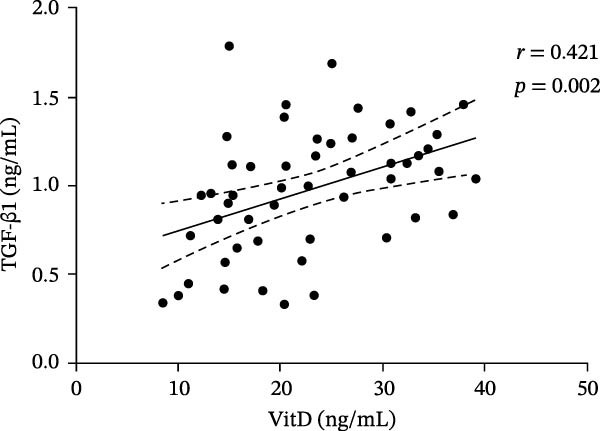
(A)

**Figure:**
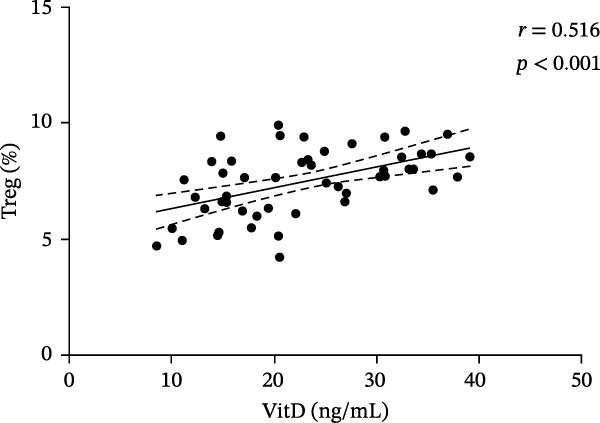
(B)

**Figure:**
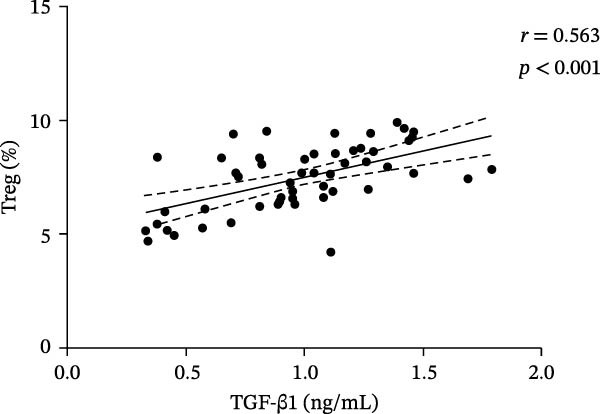
(C)

### 3.3. Correlation Between VitD, TGF‐β1, Treg Cells, and Platelets in ITP and Control Groups

In the ITP group, VitD was positively correlated with PLT count (*r* = 0.399, *p* = 0.004), TGF‐β1 and PLT count (*r* = 0.305, *p* = 0.029), and Treg cells and PLT count (*r* = 0.361, *p* = 0.009) (Figure 3). In the control group, there was no correlation between VitD and PLT (*r* = 0.130, *p* = 0.401), TGF‐β1 and PLT (*r* = −0.130, *p* = 0.402), or between Treg cells and PLT (*r* = 0.041, *p* = 0.794).

Figure 3Correlation between VitD, TGF‐β1, Treg cells, and platelet count in the ITP group. (A), (B), and (C) represent the correlation between VitD and platelet count, TGF‐β1 and platelet count, and Treg cells and platelet count, respectively. Sample size: *n* = 51.

**Figure:**
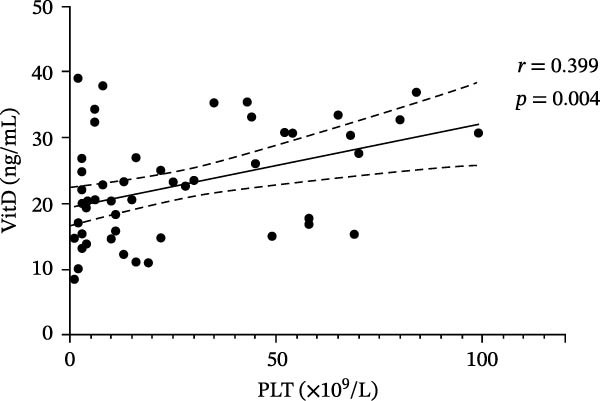
(A)

**Figure:**
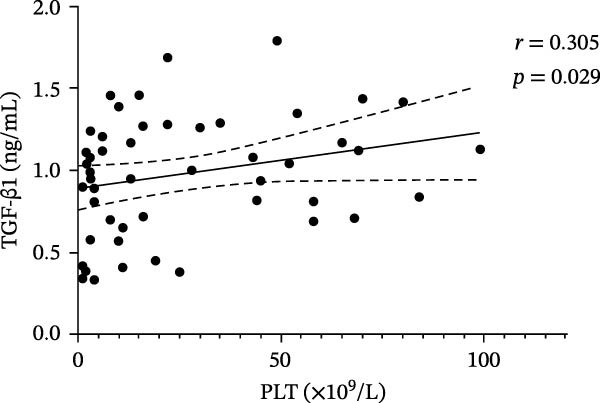
(B)

**Figure:**
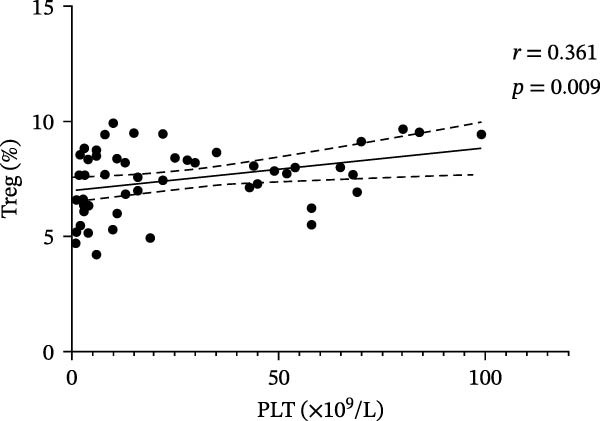
(C)

### 3.4. Correlation Between VitD, TGF‐β1, and Treg Cells in the Total Population

In order to explore the association between each factor and disease in the next step, the total population needs to be included in a group to verify whether the association exists. These results showed that there was a positive correlation between VitD and TGF‐β1 level (*r* = 0.379, *p*  < 0.001), VitD and Treg cell level (*r* = 0.360, *p*  < 0.001), and Treg cell and TGF‐β1 level (*r* = 0.580, *p*  < 0.001) (Figure 4).

Figure 4Correlation analysis of VitD, TGF‐β1, and Treg cells in the total population. (A), (B), and (C) represent the correlation between VitD and TGF‐β1, VitD and Treg cells, and Treg cells and TGF‐β1, respectively. Sample size: *n* = 95.

**Figure:**
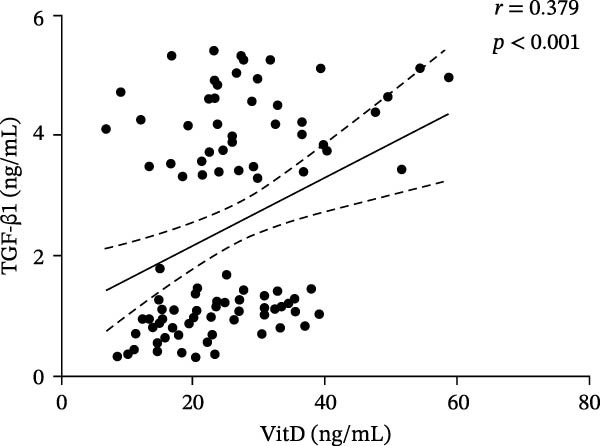
(A)

**Figure:**
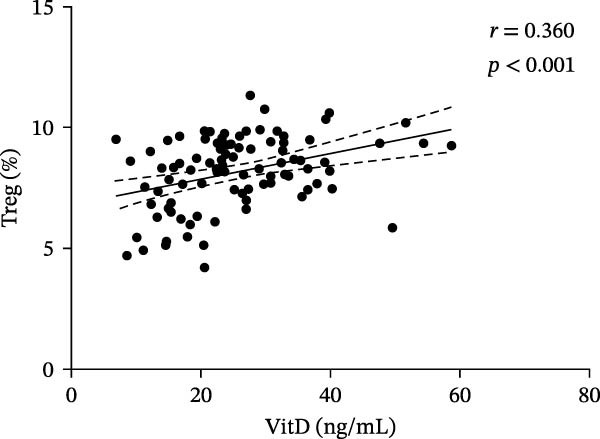
(B)

**Figure:**
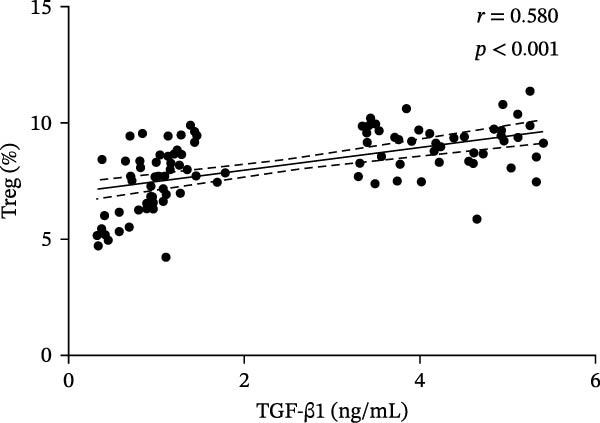
(C)

### 3.5. The Mediating Effect of TGF‐β1 and Treg Cells on the Pathogenesis of ITP Induced by VitD

In order to further explore the relationship between VitD, TGF‐β1, and Treg cells in the pathogenesis of ITP and their mediating effects, a chain‐mediated model was constructed (VitD as independent variable *X*, TGF‐β1 as intermediate M1, Treg as intermediate M2, and ITP onset as dependent variable *Y*). Multiple regression results showed that VitD significantly positively predicted TGF‐β1 (*β* = 0.056, *p*  < 0.01) and Treg (*β* = 0.029, *p* = 0.028), and TGF‐β1 significantly positively predicted Treg (*β* = 0.488, *p*  < 0.01). There was a significant negative direct effect of VitD on ITP (*β* = −0.014, *p* = 0.004). The risk of ITP decreased by 1.4% when serum VitD increased by 1 ng/mL. However, the direct effect was not significant when the intermediate variables were included (*β* = 0.001, *p* = 0.374). TGF‐β1 negatively predicted ITP (*β* = −0.283, *p*  < 0.01), while Treg did not (*β* = 0.006, *p* = 0.622) (Table 2).

**Table 2:** Regression analysis of the mediating model of TGF‐β1 and Treg cells between VitD and ITP.

Item	TGF‐β1	Treg	ITP1	ITP2
Constant	1.057 ^∗^	6.383 ^∗∗^	0.889 ^∗∗^	1.160 ^∗∗^
VitD	0.056 ^∗∗^	0.029 ^∗^	−0.014 ^∗∗^	0.001
TGF‐β1	—	0.488 ^∗∗^	—	−0.283 ^∗∗^
Treg	—	—	—	0.006
*R* ^2^	0.115	0.354	0.086	0.909
Adjust *R* ^2^	0.106	0.340	0.076	0.906
*F*‐value	12.126 ^∗∗^	25.220 ^∗∗^	8.782 ^∗^	303.528 ^∗∗^

*Note:* ITP1 indicates the direct effect of VitD on ITP; ITP2 represents the effect of VitD on ITP and the effect of each intermediary variable on ITP.

^∗^ *p*  < 0.05.

^∗∗^ *p*  < 0.01.

**Figure 5:**
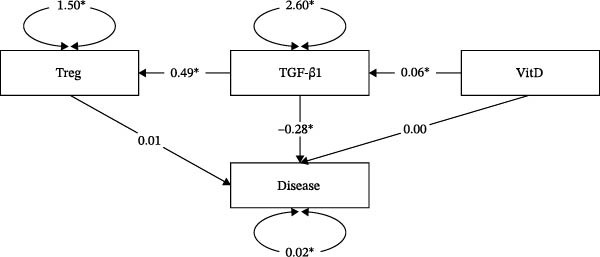
Model diagram of the mediating effect of TGF‐β1 and Treg cells in the pathogenesis of VitD and ITP.  ^∗^ *p*  < 0.05. “Disease” refers to ITP. Figures represent regression coefficients. Sample size: *n* = 95.

The results of the mediation pathway validation were as follows:

The total effect was the effect of VitD on ITP without considering the mediation variables, and the effect value was −0.014 (*p* = 0.004, 95% CI [−0.024, −0.005]), indicating that the higher the VitD level, the lower the risk of ITP, and the difference was significant. The direct effect of VitD on ITP was 0.001(*p* = 0.379, 95% CI [−0.002, 0.005]) and was not significant, indicating that the negative effect of VitD on ITP was mainly realized by the intermediary variable. The total indirect effect was the mediating effect of VitD on ITP. The value of the effect was −0.015 (*p*  < 0.001, 95% CI [−0.490, −0.138]), which indicated that the intermediary variable as a whole significantly mediated the effect of VitD on ITP. Each indirect path is as follows (Table 3 and Figure 5):

**Table 3:** Analysis of mediating effects of TGF‐β1 and treg cells between VitD and ITP.

Model effect	Effect value	SE	Bootstrap 95% CI	*z*	*p*
Total effect	−0.014	0.005	(−0.024, −0.005)	−2.963	0.004
Direct effect	0.001	0.002	(−0.002, 0.005)	0.884	0.379
Total indirect effects	−0.015	0.090	(−0.490, −0.138)	−0.173	<0.001
VitD⇒TGF‐β1⇒ITP	−0.015	0.092	(−0.500, −0.141)	−0.172	<0.001
VitD⇒Treg⇒ITP	0.000	0.009	(−0.012, 0.024)	0.018	0.986
VitD⇒TGF‐β1⇒Treg⇒ITP	0.000	0.007	(−0.009, 0.019)	0.020	0.984

*Note:* ITP, primary immune thrombocytopenia. Bootstrap 95% CI: 95% confidence interval, excluding 0 means the model is meaningful.

Abbreviation: SE, standard error.

The effect value of the VitD→TGF‐β1→ITP pathway was −0.015 (*p*  < 0.001, 95% CI [−0.500; −0.141]), indicating that VitD significantly and indirectly reduces the risk of ITP by increasing TGF‐β1 levels.

The effect value of the VitD→Treg→ITP pathway was 0 (*p* = 0.986, 95% CI [−0.012, 0.024]), indicating that the indirect effect of VitD through Treg cells was not significant, and Treg cells may not be an effective mediator.

The effect value of the VitD→TGF‐β1→Treg→ITP pathway was 0 (*p* = 0.984, 95% CI [−0.009, 0.019]) and was not significant, indicating that the pathway‐mediated effect was not significant even though TGF‐β1 affected Treg. In conclusion, the influence of VitD on ITP is mainly realized through TGF‐β1 mediation.

## 4. Discussion

ITP in children is a common bleeding disorder, mainly caused by autoimmune reactions, often accompanied by mucocutaneous hemorrhage [14]. In this study, 90.2% of the patients had this symptom, which was consistent with the characteristics of the disease. The pathogenesis of ITP is complex, involving multiple mechanisms such as complement activation, Fcγ receptor phagocytosis, T cell abnormalities, and bone marrow megakaryocyte development disorder, in addition to B cell‐mediated autoantibody destruction of platelets [15]. Although autoantibodies are critical, Vrbensky et al. [16] found that 40% of patients had no antibodies, and the antibody positive rate of newly diagnosed children in this study was only 10.2%, suggesting that non‐antibody mechanisms are also important. Cellular immune disorders in children with ITP are mainly manifested by the imbalance of CD4 + T cell differentiation; in particular, the number and function of Treg cells are the core of immune tolerance disruption [17, 18]. Treg cells maintain immune homeostasis by secreting TGF‐β1, and their dysfunction is closely related to the course of ITP [19]. As an immunomodulator, VitD can regulate the expression of TGF‐β1 and the function of Treg cells and participate in the establishment of immune tolerance, but its role in ITP is unknown [20].

VitD is a pleiotropic steroid hormone that regulates innate and adaptive immunity by inhibiting B cell proliferation and antibody production, promoting Treg/Th2 differentiation, suppressing Th1/Th17 polarization, and maintaining immune balance [21–23]. VitD deficiency is common in children with ITP [12]; in this study, only 27.45% of patients had sufficient VitD, significantly lower than healthy controls, and levels were positively correlated with platelet count, suggesting potential as a predictor of treatment response [24, 25]. Low VitD may disrupt T cell differentiation, reduce B cell inhibition, promote autoantibody production, and aggravate platelet destruction, providing a theoretical basis for combination therapy with VitD. Moreover, the correlation between VitD levels and platelet counts indicates that adequate VitD may influence disease severity and clinical manifestations, highlighting its potential as a modifiable risk factor in ITP management. Although our study could not assess the longitudinal effects of supplementation, future interventional studies are warranted to determine its therapeutic value and optimal dosing. Treg cells maintain immune tolerance by inhibiting Th1/Th17 and CD8+ T cell activation, blocking dendritic cell maturation, and secreting IL‐10 and TGF‐β1 to create an inhibitory immune environment [26, 27]. ITP patients have reduced Treg cells in blood and spleen bone marrow, resulting in immune imbalance [28, 29]. In chronic ITP, dexamethasone increased both Treg proportions and platelet counts, suggesting Treg reconstitution as a marker of treatment response [30]. In newly diagnosed patients, Treg levels were significantly lower and positively correlated with platelet count (*r* = 0.361, *p* = 0.009), supporting their role in ITP pathogenesis, although some studies report no correlation, reflecting the disease’s complexity [31]. TGF‐β1, a key immunomodulator that inhibits B cell proliferation, regulates T cell differentiation, and maintains Treg function [32, 33], was significantly lower in ITP children compared with controls and positively correlated with platelet count, suggesting that impaired TGF‐β1 metabolism is a core pathogenic mechanism. Low TGF‐β1 may impair Treg development, weaken immune regulation, and increase platelet apoptosis, exacerbating disease progression [34–36]. Thus, TGF‐β1 serves both as a pathological mediator and a potential biomarker for ITP classification and treatment response. Taken together, these findings emphasize the clinical relevance of VitD and TGF‐β1 as immunoregulatory markers and support future studies to explore VitD supplementation as a strategy to modulate TGF‐β1/Treg function and improve clinical outcomes in pediatric ITP.

The interaction of VitD, TGF‐β1, and Treg cells in ITP is still under exploration. Basic research shows that Treg cells play an immunosuppressive function by expressing the specific transcription factor Foxp3. Activated Treg cells can secrete TGF‐β1 at a high level, while TGF‐β1 promotes the induced expression of Foxp3 in peripheral immature CD4+ T cells and maintains the number and function of Treg cells [37]. Clinical observation shows that the decrease in frequency and function of CD4 + CD25 + Foxp3 + Treg in children with ITP is closely related to the decrease in serum TGF‐β1 level [38]. In vitro experiments further show that high concentration of VitD can significantly increase the expression of TGF‐β1 and promote the differentiation and function enhancement of Treg cells [39], and VitD directly binds to the Foxp3 gene promoter through its receptor VDR to induce Foxp3 expression, thus increasing the number of Treg cells [40]. These results showed that VitD level was positively correlated with the ratio of Treg and the concentration of TGF‐β1 in children with ITP, but not in the healthy control group, suggesting that the imbalance of the regulatory network of “VitD→TGF‐β1→Treg” may be the key mechanism in the pathogenesis of ITP. We hypothesize that the decrease in VitD level leads to the decrease in TGF‐β1 expression, which, in turn, reduces the number of Treg cells and the immunoregulatory ability, and forms a vicious circle of Treg reduction and TGF‐β1 reduction and finally exacerbates the immune‐mediated platelet destruction and underproduction. Mediator effect analysis further confirmed that TGF‐β1 plays an important mediating role in the pathogenesis of ITP, emphasizing its clinical and mechanistic significance as a core hub.

In order to further explore the relationship between the three factors and ITP and their mediating effects, we constructed a chain‐mediated effect model to explore the effects of VitD on the pathogenesis of ITP through TGF‐β1 and Treg cells. These results showed that the total effect of VitD on ITP was significantly negative (regression coefficient was −0.014, *p* = 0.004). When TGF‐β1 and Treg cells were included as mediators, the direct effect of VitD on ITP was no longer significant (*β* = 0.001, *p* = 0.379), indicating complete mediation. The total indirect effect was significant (effect = −0.015, *p*  < 0.001), demonstrating that the mediators play a key role in the influence of VitD on ITP. Pathway‐specific analysis showed that the VitD→TGF‐β1→ITP pathway accounted for this effect (effect = −0.015, *p*  < 0.001), indicating that VitD reduces ITP risk primarily through upregulation of TGF‐β1 rather than via a direct pathway or through Treg cells in this model. These results highlight TGF‐β1 as a central mechanistic hub in VitD‐mediated immune regulation and underscore its potential as a therapeutic target in pediatric ITP, emphasizing the importance of TGF‐β1‐focused strategies in restoring immune balance and mitigating disease risk. Although the independent (Treg → ITP) and chain (VitD → TGF‐β1 → Treg → ITP) mediation pathways were not statistically significant, this does not diminish their biological relevance. Treg cells were significantly lower in children with ITP and showed positive correlations with both VitD and TGF‐β1, supporting their role in immune dysregulation. The nonsignificance likely reflects the limited sample size and the complex, multifactorial nature of ITP, rather than an absence of effect. These results suggest that Treg cells remain a key component of the VitD/TGF‐β1 axis, contributing to immune tolerance and platelet regulation. Despite statistical limitations, the observed correlations highlight an important biological interplay among VitD, TGF‐β1, and Treg cells. Future studies with larger cohorts and more comprehensive models are needed to clarify the precise mediating role of Treg cells in the pathogenesis of ITP.

To sum up, the expression of TGF‐β1 and Treg cells decreased in children with ITP, and the levels of TGF‐β1 and Treg cells were positively correlated with platelet count, indicating that the change of Treg cell proportion and the decrease in TGF‐β1 level were closely related to the immune imbalance of primary ITP in children. The low level of VitD is common in children with ITP, and there is a positive correlation between the level of VitD and the proportion of Treg cells, the concentration of TGF‐β1, and the platelet count. The chain‐mediated model further revealed that the risk effect of VitD on ITP was mainly achieved by downregulation of TGF‐β1, while the independent mediating effect of Treg cells was not significant, which may be due to the small sample size. It is also suggested that VitD supplementation may reverse the immune imbalance by reconstructing the function of TGF‐β1 and Treg cells in the treatment of ITP.

This study has several limitations. It is a single‐center, retrospective study with a relatively small sample size, which may underestimate the independent effects of Treg cells and limit the generalizability of the findings; thus, larger multicenter studies are warranted for validation. Additionally, due to incomplete follow‐up in some patients, we were unable to measure posttreatment levels of VitD, Treg cells, and TGF‐β1, preventing assessment of their dynamic changes or direct impact on therapy response and disease chronicity. VitD levels are influenced by multiple factors, including sunlight exposure, diet, living environment, and geographical location. Although participants were from the same region with similar backgrounds, individual lifestyle differences may still affect VitD status. Future studies with optimized design and longitudinal follow‐up are needed to clarify the clinical impact of VitD and immunoregulatory pathways in pediatric ITP.

## 5. Conclusion

This study demonstrates that VitD levels are significantly lower in children with primary ITP compared to healthy controls, and this deficiency is associated with reduced levels of TGF‐β1 and Tregs. The mediation analysis reveals that the protective effect of VitD on ITP is primarily mediated through TGF‐β1, highlighting its critical role in the pathogenesis of ITP. These findings suggest that targeting the TGF‐β1/Treg pathway may offer therapeutic potential for pediatric ITP, and VitD supplementation could be a valuable adjunctive treatment to restore immune balance. Future research should focus on validating these results in larger cohorts and exploring the clinical efficacy of VitD‐based interventions.

## Author Contributions

Peiling Li, Mengru Chen, Dongju Zhao, and Shujun Li conceived of the study. Zhihang Wang, Rui Fan, and Jia Guo participated in its design and coordination. Bao Liu, Zhiyin Wang, and Yanyan Ma helped to draft the manuscript.

## Funding

This study was funded by the Joint Construction Project of Henan Province Medical Science and Technology Research Program (Grant LHGJ20200518).

## Disclosure

All authors read and approved the final manuscript.

## Ethics Statement

This study was conducted in accordance with the Declaration of Helsinki. The study was approved by the Medical Ethics Committee of the First Affiliated Hospital of Xinxiang Medical College (Approval Number EC‐023‐222). Due to the retrospective nature of the study and the use of anonymized patient information, informed consent was waived with the approval of the Ethics Committee of Xinxiang Medical College. All methods were carried out in accordance with relevant guidelines and regulations.

## Conflicts of Interest

The authors declare no conflicts of interest.

## Data Availability

All data generated or analyzed during this study are included in this published article.
